# Sex Trafficking of Women and Girls in Canada: A Scoping Review of the Scholarly Literature

**DOI:** 10.1177/15248380221094316

**Published:** 2022-05-20

**Authors:** Evelyn Hodgins, Julie Mutis, Robin Mason, Janice Du Mont

**Affiliations:** 1 Women's College Research Institute, 7985 Women’s College Hospital, Toronto, ON, Canada

**Keywords:** Canada, Intersectionality, Review, Sex Trafficking, Women

## Abstract

Sex trafficking has been identified as a prominant health and human rights concern in Canada. However, there has been little empirical research on the topic and existing studies are largely found within the grey literature. This review sought to identify and summarize the current scholarly literature about sex trafficking of women and girls in Canada. We identified empirical studies using a keyword search in ProQuest, Web of Science, and Scopus. Eligible articles were published in English in 2000 or later, included a focus on women victim/survivors, and analyzed human/participant data. Only 14 studies met eligibility criteria. Most studies were qualitative, based on interviews or focus groups primarily with stakeholders, and set in the province of Ontario. Key findings highlighted challenges in conceptualizing sex trafficking centered largely around issues of coercion and consent. Pathways into trafficking (economic displacement, past abuse, and broken ties with family and community) and gaps and barriers in anti-trafficking responses (narrow or conflicting definitions, stigmatization and criminalization of sex work, and a lack of accessible or appropriate services) particularly impacted Indigenous, im/migrant, and other marginalized women and girls. There is a pausity of empirical studies on sex trafficking in Canada and this has implications for the development of data-driven policies and protocols. Further research should seek to highlight the voices of survivors and impacted communities and evaluate strengths and limitations of Canadian anti-trafficking interventions.

Sex trafficking has been identified as a pervasive health and human rights issue and is a prominent topic of global and Canadian domestic policy development. Since signing the 2000 United Nations *Protocol to Prevent, Suppress, and Punish Trafficking in Persons, Especially Women and Children* (hereinafter *The Protocol*, UNGA, 2000), which addresses trafficking of all forms including sex and labor trafficking, Canada has actively pursued anti-trafficking action through legislation, programs, and funding, including the recent *National Strategy to Combat Human Trafficking: 2019–2024* (Goodale, 2019). After two decades of investment, data from the Uniform Crime Report has shown a general increase in trafficking-related charges, with charges in 2019 increasing by 44% from the previous year (Ibrahim, 2021). However, accurate prevalence data on human trafficking is notoriously difficult to collect, and Ibrahim (2021) has noted that the Uniform Crime Report data may reflect changes in policy and increased resources for investigation, as opposed to increased trafficking incidence. Moreover, the data are not disaggregated by trafficking type, leaving significant gaps in our knowledge of sex trafficking.

Despite the lack of evidence on the scope of sex trafficking, those in the field have some sense of who is at the highest risk. There is general agreement that sex trafficking predominantly impacts women and children, especially those who experience other intersections of oppression and economic vulnerabilities (United Nations Office of Drugs and Crime [UNODC], 2021). A history of traumatic childhood experiences, especially sexual abuse, is associated with a substantially increased risk of sex trafficking victimization (De Vries & Goggin, 2020; Franchino-Olsen, 2021). There are clear health consequences of being sex trafficked, including mental health impacts such as depression, post-traumatic stress disorder, suicidality and substance use, and sexual health impacts such as sexually transmitted infections (STIs) and pregnancy (Barnert et al., 2017; Le et al., 2018).

## Canada’s Legal and Policy Landscape

*The Protocol* presented a contemporary definition of trafficking in persons, which specified three components of the crime: action, means, and purpose. The definition of human trafficking laid out in *The Protocol* reads as follows:

The recruitment, transportation, transfer, harbouring or receipt of persons, by means of the threat or use of force or other forms of coercion, of abduction, of fraud, of deception, of the abuse of power or of a position of vulnerability or of the giving or receiving of payments or benefits to achieve the consent of a person having control over another person, for the purpose of exploitation. Exploitation shall include, at a minimum, the exploitation of the prostitution of others or other forms of sexual exploitation, forced labour or services, slavery or practices similar to slavery, servitude or the removal of organs. (Section 3.a)

Shortly after signing *The Protocol,* Canada introduced measures to criminalize human trafficking in pursuit of meeting their obligations under the agreement. Sections of the 2001 *Immigration and Refugee Protection Act* (IRPA) prohibited the facilitation of bringing persons into Canada “by means of abduction, fraud, deception or threat of force or coercion,” (IRPA, 2001). In 2005, additions were made to Canada’s *Criminal Code* that addressed international and domestic trafficking (Government of Canada, 2021). Both pieces of legislation were applicable to all forms of trafficking in humans but trafficking for the purpose of sexual exploitation was considered an aggravating factor that increased penalties.

## Canadian Research and Reviews

Timoshkina and McDonald (2009) performed the first synthesis (to their knowledge and ours) of the empirical literature on sex trafficking in Canada. They found that the majority of the literature was focused on policy analysis or conceptual papers, not empirical evidence. Studies in their review came largely from the gray literature and used qualitative methods. The authors limited their meta-synthesis of the qualitative data to those articles focusing on transnational sex trafficking into Canada, the most common focus in the identified literature. They found that the variation in definitions used and the tendency to frame the problem primarily through a criminal justice lens, as opposed to centering the rights and needs of trafficking victim/survivors, were issues identified in the reviewed studies.

Since 2009, notable organizational reports, such as the *Task Force on Trafficking of Women and Girls in Canada* reports from 2013 and 2014, commissioned by the Canadian Women’s Foundation, were completed. These reports, based on summaries of the existing literature, surveys, and interview data, highlighted the importance of recognizing domestic sex trafficking and the disproportional victimization of Indigenous women and children (Barrett, 2013; Native Women’s Association of Canada [NWAC], 2014). Two more recent reviews have focused on the trafficking and the commercial sexual exploitation of children (CSEC); one considered children’s exposure to sex trafficking, sexual exploitation, and community-based violence in Canada (Kimber & Ferdossifard, 2021), and the other, set in Canada and the United States, recruitment and entrapment pathways in the sex trafficking of minors (Baird & Connolly, 2021). Both reviews found few Canadian studies meeting their criteria.

To our knowledge, there has been no recent review on the scope of sex trafficking literature in Canada that has examined adolescent and adult populations since Timoshkina and McDonald (2009) and none synthesizing the full scope of the scholarly literature. Thus, we undertook a systematic scoping review to determine what is currently known in the Canadian scholarly literature on sex trafficking of adolescent girls and women, the primary victims of sex trafficking.

## Methods

### Search Strategy

A search strategy was developed and the search was performed in ProQuest, Scopus, and Web of Science (see Appendix A).

The search strategy included terms commonly equated to or overlapping with sex trafficking in the literature such as “CSEC,” “forced prostitution,” and “modern slavery.” We also combined concepts from *The Protocol* and Canada’s *Criminal Code* definitions of sex trafficking such as “coercion” and “sex work” to retrieve those articles that used the terms together. Terms such as “women,” “girls,” “youth,” and “minor” were used, reflecting our population of focus. All terms were then combined with the Boolean OR, and the Boolean AND with “Canada” and locales within Canada. Filters were applied to limit the results to English-language scholarly journals published in the year 2000, the year that *The Protocol* was signed, or later (Web of Science did not have a scholarly journals filter, see Appendix B).

The final search and retrieval were performed on June 24th, 2021, yielding 514 records. All records were imported to Covidence and, after removing duplicates, 346 unique records remained.

### Article Selection

Article inclusion and exclusion criteria were developed throughout the planning and searching phases of the review, starting broadly and then narrowing criteria as the search was refined (Arksey & O’Malley, 2005). Screening was performed independently by two reviewers using a screening tool which was developed, tested, and refined for clarity. To be included during title/abstract screening, the articles must have: been published in a peer-reviewed journal, focused on sex trafficking, been set in Canada, and used an empirical research design. Empirical research was limited to studies which included human or “participant” data. Articles in which it was difficult to determine whether the victim/survivors of sex trafficking were women and girls were included; however, those with an explicit focus on males or an all-male sample were excluded. Also excluded were previous systematic reviews. Articles for which inclusion/exclusion could not be determined by title/abstract screening were included for full-text review.

At the full-text review stage, any remaining articles that did not meet the inclusion criteria were excluded. Also excluded were articles that did not identify the issue under investigation as “sex trafficking” even if there were features consistent with that definition (e.g., Nixon et al., 2002); this was done to avoid the possibility of incorrectly labeling something as sex trafficking. Additionally, one non-English language article was identified and excluded at this stage.

**Figure 1.**
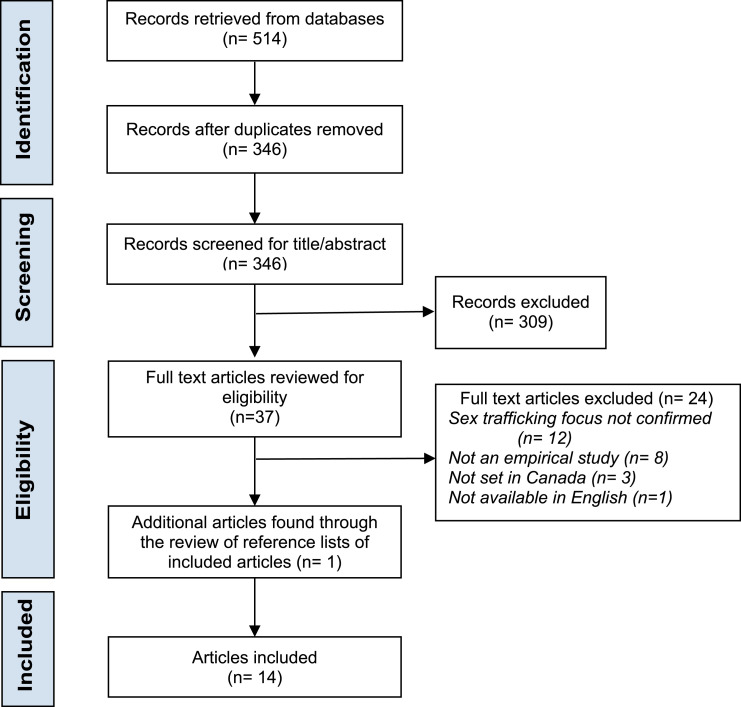
PRISMA flow diagram.

Most conflicts were resolved by discussion between two reviewers (EH, JM); however, those for which agreement could not be reached, were brought to the third and fourth reviewers (JDM, RM) for a final decision. A total of 309 articles were excluded through title/abstract screening, and twenty-four articles were excluded for not meeting eligibility criteria in the full-text review. One additional article was found through the review of the reference lists of included articles. A final 14 articles were included in the review (see Figure 1).

### Data Extraction and Synthesis

Data from each of the 14 articles were extracted in Covidence independently by two reviewers and consensus was reached through discussion (see Table 1). Two of the included articles were based on the same research but focused on different findings and reported different study purposes; data extraction was completed for both articles (McDonald & Timoshkina, 2004, 2007). Findings from each of the studies were analyzed and synthesized into themes. As a quality assessment of the reviewed evidence is not typically within the purview of a scoping review, one was not performed (Arksey & O’Malley, 2005).

**Table 1.** Study Characteristics and Findings.

Author, year, and setting	Purpose	Sex trafficking definition	Sample & Demographics	Methods	Key findings	Stated limitations
Baird, K., McDonald, K. P., & Connolly, J. (2019) Greater Toronto Area (GTA), Ontario	Increase understanding of the elevated risk of sex trafficking among child welfare-involved youth	Paraphrase of the Protocol to Prevent, Suppress and Punish Trafficking in Persons Especially Women and Children [UNGA, 2000]^a^	223 women and female youth identified as victims/survivors of sex trafficking: White (57%), South Asian (17.9%), Black/African Canadian (16.1%), Hispanic, Asian, and Indigenous (5%)	Secondary analysis of police and child welfare data	Child welfare-involved victims/survivors were recruited into sex trafficking younger, more often via grooming, and had a higher incidence of past child maltreatment and alcohol, cocaine, and crystal meth use compared to non-child welfare-involved victims Domestic sex trafficking was more common (81.8%) than international, especially among child welfare-involved victims/survivors (98% domestic)	Findings may not be representative or generalizable to other settings as only included police-involved women and female youth in GTA Data missing for many child welfare-involved cases
De Shalit, A., van der Meulen, E., & Guta, A. (2020) Ontario	Explore how sex trafficking is represented as a public health problem, the assumptions that underlie it, and its discursive and practical effect	No definition provided	22 service providers from organizations involved in anti-trafficking response (e.g., health, social, and legal services) that received government funding	In-depth semi-structured interviews	Majority of service providers conflated sex work and sex trafficking, framed both as public health issues to be solved through self-regulation and law enforcement	Findings may not be representative of all government-funded anti-trafficking agencies, and agencies receiving funding from other sources were not included in the sample May not be generalizable to other provinces
Gow, G. A., Barlott, T., Quinn, K., Linder, J., et al. (2015) Edmonton, Alberta	Consider how text messaging from anti-trafficking/anti-sexual exploitation organizations might be used to initiate outreach with individuals soliciting through the adult services section of the Website, Backpage.com	No definition provided	565 phone numbers linked to advertisements for sexual services on Backpage.com	Community-based participatory research and program evaluation	14% of recipients of outreach text messages responded 77.1% of responses were neutral (e.g., “no thanks”), 14.2% were negative (e.g., “don’t text me that bull****”) and 7.7% were positive (e.g., “Thank you its [sic] an amazing thing you’re doing”) Some instances in which new service users reported having received the text messages Two-way messaging is a promising practice for providing information and social support	Analysis did not systematically examine impacts Limited generalizability of findings
Hanley, J., Oxman-Martinez, J., Lacroix, M., & Gal, S. (2006) Vancouver, British Columbia Toronto, Ontario Montreal, Quebec, and Winnipeg, Manitoba	Deepen understanding of the characteristics and needs of victims of human trafficking, document community-based services and service gaps for victim/survivors	Protocol to Prevent, Suppress and Punish Trafficking in Persons Especially Women and Children [UNGA, 2000]^a^	40 stakeholders from organizations with direct experience with sex and/or labor trafficking victims (e.g., victim services, health services, immigration, and refugee services, activist groups)	Interviews	Sex trafficking deemed the most dangerous form of trafficking for women Sex trafficking victims/survivors experience low or no pay, long hours, physical and sexual abuse, and untreated health conditions (STIs, mental health, addiction) Conceptions of sex trafficking varied Economic opportunity strongest motivator for migration and subsequent sex or labor trafficking	None
Kaye, J., Winterdyk, J., & Quarterman, L. (2014) Calgary, Alberta	Explore stakeholders’ understandings of the local patterns and trends of trafficking, and their perceptions of existing counter-trafficking responses	No definition provided	53 stakeholders from organizations involved in Alberta’s anti-trafficking response, 18 of whom also participated in focus groups (e.g., government officials, law enforcement personnel, and representatives from social service agencies)	Survey and focus groups	44% of survey respondents believed they had encountered a victim of any form of trafficking Agreement that females and Indigenous persons are overrepresented among sex trafficking victims/survivors Barriers for Indigenous peoples accessing help included the lack of culturally appropriate awareness training and a focus on criminal justice responses to trafficking Labor exploitation was believed to be more common than sexual exploitation in international trafficking	Comparisons of different trafficking types and demographics not based on quantitative prevalence data
Matheson, C. M,. & Finkel, R. (2013) Vancouver, British Columbia	Examine stakeholder perceptions of the potential linkages between sex trafficking and the Vancouver Winter Olympics in 2010, and the preventative measures implemented in relation to the event	Protocol to Prevent, Suppress and Punish Trafficking in Persons Especially Women and Children [UNGA, 2000]^a^	23 stakeholders with expertise on relevant policies, sex trafficking, or the sex industry in Vancouver (e.g., government officials, law enforcement personnel, and representatives from social service agencies)	Semi-structured in-depth interviews	Frontline workers providing victim services, connected to the local sex trade or with trafficking investigative experience, largely had abolitionist perspectives on sex work and saw the games as a catalyst, whereas those connected to the sex worker rights movement or representing a human rights perspective on trafficking, largely did not agree Fragmented preventative measures around the games were in part attributed to these opposing positions	Changes in sex trafficking incidence could not be determined due to a lack of reliable baseline data
McDonald, L., & Timoshkina, N. (2007) Toronto, Ontario	Determine how health and social service needs of sex-trafficked women could best be met given their day-to-day experiences	Chew [1999].^b^ Wijers & Lap-Chew [1997].^c^	20 women identified as victim/survivors of sex trafficking: Canadian women (*n* = 2) were prior university students Eastern European women (*n* = 18) had all finished high school, and at least half had graduated from university or vocational schools 15 service providers (e.g., health, legal, and social services); and 15 key informants with knowledge of the population (e.g., law enforcement, managers in the sex industry, former sex workers)	Semi-structured in-depth interviews	Experiences of victim/survivors often involved both coercion and consent, contrary to the service providers’ perceptions Barriers to accessing medical and social services included a lack of knowledge about services, stigmatization, and fear of arrest or deportation Victim/survivors preferred to access services from non-specialized agencies and wanted services pertaining to education, employment, and immigration	Victim/survivors included only those who were still being trafficked and may not be representative of those who left Information provided by victim/survivors was sometimes vague and guarded
McDonald, L., & Timoshkina, N. (2007) Toronto, Ontario	Understand the working lives of Eastern/Central European women sex trafficked into Canada	Chew [1999].^b^ Wijers & Lap-Chew [1997].^c^	20 women identified as victim/survivors of sex trafficking: Canadian women (*n* = 2) were prior university students Eastern European women (*n* = 18) had all finished high school, and at least half had graduated from university or vocational schools 15 service providers (e.g., health, legal, and social services), and 15 key informants with knowledge of the population (e.g., law enforcement, managers in the sex industry, former sex workers)	Semi-structured in-depth interviews	Seeking economic opportunity and fleeing war-like political situations were motivations for migration; most women were subjected to some level of deception and debt-bondage in their recruitment Those brought to Canada under false pretenses experienced the most control, exploitation, and threats to their safety There was variation in the level of choice and agency the women demonstrated (e.g., some women stayed in sex trafficking due to a lack of employment opportunities and fear of deportation)	None
Millar, H., & O’Doherty, T. (2020) All Canadian regions	Investigate the nature of prosecuted cases involving sex or labor trafficking charges in Canada and the presence of racial and gender bias and sensationalism in the identification and prosecution of these cases	No definition provided	87 prosecuted cases involving sex or labor trafficking charges between 2001 and 2018 and the corresponding media reports where available: Indigenous victim/survivors in 2 cases and Black/ African Canadian victim/survivors in “2 or 3” cases identified in court proceedings	Secondary analysis of published court proceedings and mainstream media (mostly print) coverage	48.8% of 87 prosecuted cases resulted in trafficking-specific convictions; in the remaining prosecutions, trafficking-specific charges were withdrawn 80 of the 87 cases (92%) reflected alleged domestic sex trafficking; one involved alleged transnational sex trafficking; one involved alleged transnational trafficking for marriage; the remaining 5 involved alleged transnational labor trafficking Negligible evidence of organized criminal involvement in court proceedings data Racial and gender bias and associated sensationalism exist in criminal justice responses to trafficking and could impact reporting and prosecution	Findings are tentative as based on textual examination of court proceedings and media coverage including interpretation of media images Limited Canadian trafficking data available to contextualize findings
Morselli, C., & Savoie-Gargiso, I. (2014) Montreal, Quebec	Explore the dynamics of control in a “prostitution ring” involved in suspected sex trafficking	Authors’ definition: “Trafficking occurs, according to Canadian and American law, when the relationship [between a pimp and sex worker] is forged under coercion, deception, or physical force or when it involves minors.”	142 members of a “prostitution ring” including “pimps” and sex-trafficked women	Social network analysis and thematic analysis of electronic police surveillance data	Many women in the network met legal criteria for sexually trafficked persons The network was clustered around pimps and high-status sex workers who participated in its management While manipulation and coercion were observed, collaborative relationships between “pimps” and some women who had been trafficked into the network as minors demonstrated nuanced dynamics of control	None
Nagy, R., Snooks, G., Quenneville, B., Chen, L. et al. (2020) Northeastern Ontario	Identify the needs, gaps, and barriers to services for sex-trafficked women, especially Indigenous women, in Northeastern Ontario to inform a provider toolkit and recommend social policies	No definition provided	5 individuals with lived experience in sex trafficking or the sex trade or an affected family member and 160 service providers (e.g., health, social, and Victim Services): 60/165 were Indigenous or attending on behalf of Indigenous organizations	Full-day participatory action research workshops	Gaps in trafficking response include a lack of service funding, mental and physical healthcare, affordable long-term housing, accessible, and acceptable services for Indigenous women and Francophone populations, and intersectoral communication and collaboration Additional barriers to accessing help included the criminalization of sex work and geographic inaccessibility	None
Olson-Pitawanakwat, B., & Baskin, C. (2021) Toronto, Ontario	Highlight the experiences and knowledge of Indigenous women, Two Spirit, and trans women who have experienced sex trafficking, and the professionals who provide social services	No definition provided	5 victims/ survivors of sex trafficking: Cisgender women (*n* = 3), Two Spirit (*n* = 2), and/or trans women (*n* = 1),All Indigenous 3 social service providers who support Indigenous victims/survivors of sex trafficking: All Indigenous	Interviews using a participant-led storytelling format	Generational trauma and the impact of colonization contribute to conditions that put Indigenous women, Two Spirit, and trans women at risk of sex trafficking Indigenous-led preventative measures and exit and healing initiatives are needed	Small sample size limits generalizability
Pashang, S. (2018) Greater Toronto Area, Ontario	Explore the mental health impacts of sex trafficking on illegalized female trafficked youth	Paraphrase of the *Criminal Code* of Canada [Raaflaub, 2006]^d^ Immigration and Refugee Protection Act [IRPA, 2001].^e^	31 female youth and women under the age of 29 who self-identified as victims/survivors of sex trafficking	Survey and interviews	Majority were recruited in their home countries and deceived about either the nature of work or the working conditions Many arrived in Canada legally, but were now at risk of deportation and lived and worked “underground” to avoid Canadian authorities Frequently reported negative mental health symptoms included fear (83%), anxiety about something bad happening (90%), sleeping problems and nightmares (88%), flashbacks (84%), depression (80%), sadness (77%), and self-harm or suicidal ideation (77%) Nearly all self-identified as having a sexually transmitted infection	Only reflected the experiences of those who self-identified as sex trafficking victim/survivors on the survey and volunteered to participate in follow-up interviews
Sethi, A. (2007) All Canadian regions	Gather insights to aid in distinguishing between sex work and sex trafficking and identify key issues in domestic trafficking of Indigenous girls	Protocol to Prevent, Suppress and Punish Trafficking in Persons Especially Women and Children [UNGA, 2000]^a^	18 stakeholders (e.g., service providers, researchers, program coordinators, or directors) working in the area of countering sexual exploitation in Canada	Semi-structured interviews	Intergenerational trauma, impact of residential schools, and ongoing interpersonal and structural racism, identified as root causes of sex trafficking and exploitation of Indigenous girls and women Poverty, lack of opportunity, and family violence are linked to displacement to cities and vulnerabilities exploited by traffickers In some cases, police are reluctant to address the issue in Indigenous communities	Findings not necessarily reflective of the views of trafficked girls No distinguishing between first Nations, Inuit, and Metis girls International trafficking not included

^a^The recruitment, transportation, transfer, harboring or receipt of persons, by means of the threat or use of force or other forms of coercion, of abduction, of fraud, of deception, of the abuse of power or of a position of vulnerability, or of the giving or receiving of payments or benefits to achieve the consent of a person having control over another person, for the purpose of exploitation. Exploitation shall include, at a minimum, the exploitation of the prostitution of others or other forms of sexual exploitation, forced labor or services, slavery or practices similar to slavery, servitude or the removal of organs [UNGA, 2000].

^b^Trafficking in Women: all acts involved in the recruitment and/or transportation of a woman within and across national borders for work or services by means of violence, or threat of violence, abuse of authority or dominant position, debt bondage, deception, or other forms of coercion [Chew, 1999].

^c^Forced Labor and Slavery-like Practices: The extraction of work or services from any woman or the appropriation of the legal identity and/or physical person of any woman by means of violence or threat of violence, abuse of authority or dominant position, debt bondage, deception or other forms of coercion [Wijers & Lap-Chew, 1997].

^d^*Criminal Code* offenses relevant to trafficking in persons include kidnapping, extortion, forcible confinement, conspiracy, controlling or living off the avails of prostitution, as well as organized crime offences [Raaflaub, 2006].

^e^No person shall knowingly organize the coming into Canada of one or more persons by means of abduction, fraud, deception, or use or threat of force or coercion [IRPA, 2001].

## Results

### Study Characteristics

#### Purpose

Many (*n* = 8) of the 14 studies were exploratory in nature with aims related to identifying sex trafficking patterns, key issues, and barriers to responses, and/or highlighting the experiences and needs of victim/survivors of sex trafficking. Three studies explored how stakeholders conceptualized the issue of sex trafficking and sex trafficking responses. Four studies had more defined aims including to compare characteristics and experiences of sex trafficking victim/survivors with past child welfare involvement to those without, to evaluate the feasibility and success of text messaging as an outreach intervention for victims/survivors of sex trafficking and sexual exploitation, to investigate the features, outcomes, and media representation of sex trafficking cases, and to analyze the dynamics of control in a “prostitution ring” under investigation for sex trafficking.

#### Definition of Sex Trafficking

Four of the 14 studies used the human trafficking definition developed for *The Protocol* to describe the issue of sex trafficking. Other definitions provided were those from Canada’s *Criminal Code* and the *IRPA* (*n* = 1) and alternative definitions presented in the scholarly literature, which represented the potential for coercion to be present in the recruitment, movement, and working conditions of trafficked victim/survivors (*n* = 2). Six articles in this review were not guided by any legal or scholarly definition, while one referred generally to Canadian and American law without citing a particular definition.

#### Setting

Of the 14 studies, 7 were set in Ontario, Canada’s largest province, with 5 of these specifically set in Toronto or the Greater Toronto Area (GTA). Other settings included Alberta (*n* = 2), British Columbia (*n* = 1), and Quebec (*n* = 1). Three studies included data collected from multiple cities or regions across Canada.

#### Sample & Demographics

Nine of the included studies included stakeholder participants from law enforcement, government agencies, activist groups, health, legal, or social service fields. A total of 334 stakeholders are represented across the studies. Two studies provided demographic information about the stakeholders. All three service providers in Olson-Pitawanakwat and Baskin (2021) were Indigenous. In Nagy et al. (2020), 60 of the 165 participants (5 of whom had lived experience relevant to sex trafficking or the sex industry) were either Indigenous or represented Indigenous-specific agencies.

Across five studies (Baird et al., 2019; McDonald & Timoshkina, 2004, 2007; Olson-Pitawanakwat & Baskin, 2021; Pashang, 2018) there were 279 victim/survivors of sex trafficking included, with the majority (*n* = 223) coming from a single study using law enforcement and child welfare files (Baird et al., 2019). Fifty-six of the 279 were participants in studies collecting primary data. An additional four studies included victim/survivor data from which a total could not be determined (Gow et al., 2015; Millar & O’Doherty, 2020; Morselli & Savoie-Gargiso, 2014; Nagy et al., 2020). Five studies included demographic data about victim/survivors. At least five Indigenous victim/survivors participated in interviews (Olson-Pitawanakwat & Baskin, 2021), but Indigenous women, girls, and Two-Spirit people represented less than 5% of the police and child welfare document-derived sample (Baird et al., 2019), and were identified in only two out of 87 prosecuted trafficking cases (Millar & O’Doherty, 2020). Black, South Asian, Hispanic, Asian, and Eastern European women were represented within some of the primary data (Baird et al., 2019; McDonald & Timoshkina, 2004, 2007) and Black/African Canadian women were identified in “two or three” prosecuted trafficking cases (Millar & O’Doherty, 2020, p. 35). Victim/survivors in the McDonald and Timoshkina (2004, 2007) studies had all finished high school and had completed some level of post-secondary education. Only one study described participants by their gender identity: of the five-person sample, two were Two-Spirit and one was a trans woman (Olson-Pitawanakwat & Baskin, 2021).

#### Methods

Three of the 14 studies performed secondary analyses of data from law enforcement files (*n* = 2), court transcripts (*n* = 1), and/or child welfare records (*n* = 1).

Eleven of the 14 studies collected primary data. One of these collected data on the reach of and response to a pilot outreach program, including the number of text messages received in response to outreach texts and the nature of responses. The remaining studies that collected primary data used interviews, focus groups, or participatory action research workshops (*n* = 10), and two of these studies also collected survey or questionnaire data (*n* = 2).

### Themes

Due to the exploratory and qualitative nature of many of the 14 included studies, their findings covered a broad range of topics. Findings were synthesized into the following seven themes across studies: conceptualizing sex trafficking (*n* = 9), trafficking types (*n* = 4), pathways into sex trafficking (*n* = 8), living and working conditions while being sex trafficked (*n* = 4), needs of victim/survivors (*n* = 5), gaps and barriers in anti-trafficking responses (*n* = 11), and effectiveness of interventions (*n* = 1).

#### Conceptualizing Sex Trafficking

A major theme identified in 9 of the 14 included articles was the tension in conceptualizing sex trafficking. Six studies had the aim of investigating or clarifying conceptions of sex trafficking and associated responses to it (De Shalit et al., 2020; Kaye et al., 2014; Matheson & Finkel, 2013; Millar & O’Doherty, 2020; Morselli & Savoie-Gargiso, 2014; Sethi, 2007). Three did not initially intend to explore the issue but tensions in defining sex trafficking featured prominently in their findings (Hanley et al., 2006; McDonald & Timoshkina, 2004, 2007). These studies revealed that the conceptualization of sex trafficking cannot be explored without addressing the conceptualization of sex work, particularly views on criminalization versus legalization/decriminalization and understandings of coercion and consent in the sex industry.

De Shalit et al. (2020) investigated the conceptualization of sex trafficking among representatives from agencies providing health, social, or legal services to sex trafficking victim/survivors; they found that most did not see consent or agency existing within the sex industry, and thus considered all sex work as trafficking. Matheson and Finkel (2013) found similar confusion around sex trafficking and sex work was present among some service providers in Vancouver, British Columbia. Sex-worker rights activists among their participants argued that while violence and force can exist in the sex industry, these experiences exist on a continuum (Matheson & Finkel, 2013). One law enforcement representative noted that even in cases of sex trafficking, the level of force used by traffickers varies (Matheson & Finkel, 2013).

Morselli and Savoie-Gargiso (2014) and McDonald and Timoshkina’s (2004, 2007) findings support the notion of a coercion-consent continuum in sex trafficking experiences. While the participants in these studies met definitions for sex trafficking, their experiences reflected both choice and coercion and were inconsistent with the “Madonna/Whore,” dichotomy described by service provider participants (McDonald & Timoshkina, 2004). Sethi (2007) asserted that while the difference between sex trafficking and sex work lies in the absence or presence of choice, choice is restricted by the impacts of colonization that have left Indigenous women with limited employment opportunities and trauma associated with the legacies of residential schools and other assimilation efforts. One of the key informants in this study is quoted as saying, “it is rare that Aboriginal girls or women of color experience sex work. They are often trafficked for power and control, and coerced into prostitution for their survival needs,” (p. 59).

Three studies identified that legal definitions and the criminal justice response to sex trafficking favor sensationalized conceptions of sex trafficking, where agency and consent are entirely absent, and there is an identifiable “ideal victim” (Hanley et al., 2006; Kaye et al., 2014; Millar & O’Doherty, 2020). Service providers and advocates supporting victim/survivors and law enforcement representatives expressed difficulty using legal definitions of trafficking in their work due to their inability to capture complexity in victim/survivors’ demonstration of agency (Hanley et al., 2006; Kaye et al., 2014). Several service providers with experience working with sex and/or labor trafficking victim/survivors reported that they prefer to use the *Global Alliance Against Traffic in Women, 2001* definition, as it distinguishes between conditions during recruitment, transit, and destination phases of trafficking, which can all involve coercion; others preferred not to use a specific definition at all and instead considered each situation to be unique (Hanley et al., 2006).

#### Trafficking Types

Four studies included data about the occurrence of different trafficking types in Canada based on perceptions of stakeholders, such as service providers and government representatives, working in the area of sex trafficking (Kaye et al., 2014; Matheson & Finkel, 2013), analyses of law enforcement and child welfare data (Baird et al., 2019), or court proceedings from trafficking cases (Millar & O’Doherty, 2020). It was noted in three studies that the emphasis on international sex trafficking in media coverage and trafficking policy may be sensationalized and not reflective of trafficking patterns (Kaye et al., 2014; Matheson & Finkel, 2013; Millar & O’Doherty, 2020). Millar and O’Doherty’s (2020) investigation of the outcomes of trafficking charges reported by police found that, compared to 937 police-reported charges, only 87 were actually prosecuted and just 48.8% of those cases resulted in trafficking-specific convictions; 92% of these cases reflected domestic sex trafficking charges while only 7% involved cross-border trafficking allegations. Of these transnational cases, the majority addressed trafficking for the purpose of labor exploitation and only one involved charges for alleged sex trafficking (Millar & O’Doherty, 2020). Similarly, Baird et al., (2019) found that the majority (81.8%) of victims/survivors identified by police in the GTA were living in Canada at the time of their recruitment. Additionally, several frontline workers representing a range of agencies involved in Alberta’s anti-trafficking response, including one law enforcement representative, expressed that, in their experience, trafficking for labor exploitation was more common than sex trafficking among cross-border cases of trafficking (Kaye et al., 2014).

Matheson and Finkel (2013) explored perceptions of stakeholders with relevant expertise regarding the likelihood of the 2010 Vancouver Olympic Games being a catalyst for sex trafficking in the area. They found agreement among several stakeholders, with roles in advocacy, human trafficking investigation, and service provision, that international trafficking was unlikely due to the high cost and strict border controls. According to these stakeholders, if sex trafficking were to occur in response to an increased demand for sex work in the region, it would be within Canadian borders (Matheson & Finkel, 2013).

#### Pathways into Sex Trafficking

Eight of the 14 studies explored pathways into sex trafficking situations and found that they were largely tied to systemic inequalities including the exploitation of immigrants/migrants (Hanley et al., 2006; McDonald & Timoshkina, 2004, 2007; Pashang, 2018), the impacts of colonization (Nagy et al., 2020; Olson-Pitawanakwat & Baskin, 2021; Sethi, 2007), and child welfare involvement (Baird et al., 2019). Hanley et al. (2006), McDonald and Timoshkina (2004, 2007) and Pashang (2018) focused on international trafficking or sex trafficking of immigrant/migrant women and girls. Hanley et al. (2006) reported data from frontline service providers and advocates who worked with sex and/or labor trafficking victim/survivors; participants all agreed that the most common motivator leading to migration and subsequent trafficking was economic opportunity. This was consistent with the findings from McDonald and Timoshkina (2004, 2007) and Pashang (2018) who interviewed immigrant/migrant victim/survivors. Both McDonald and Timoshkina (2004, 2007) and Pashang (2018) found that almost all of their victim/survivor participants had been deceived in some way about the nature of their employment as a part of their recruitment. While some were deceived regarding the industry they would be working in once they arrived in Canada, others knew that they would be working in the sex industry but had been lied to about the legality of the work and the income it would provide (McDonald & Timoshkina, 2004, 2007). Once in Canada, those who had been compelled to come under completely false pretenses had their passports taken to restrict their movement and options (McDonald & Timoshkina, 2007). Traffickers also recruited by exploiting the financial need of immigrant/migrant women without documentation by offering them work illegally (Hanley et al., 2006; Pashang, 2018). Several women and adolescent girls revealed that they had originally arrived in Canada with a work visa and had only been trafficked after their employers ended their contract and their visas were no longer valid (Pashang, 2018).

The ongoing impacts of colonization, such as a lack of economic opportunity in home communities, generational trauma from residential schools, displacement of children into child welfare, and subsequent breakdown of community ties, were identified in three studies of the sex trafficking pathways of Indigenous women (Nagy et al., 2020; Olson-Pitawanakwat & Baskin, 2021; Sethi, 2007). Indigenous victim/survivors in Olson-Pitawanakwat and Baskin (2021)’s study linked their experiences of sex trafficking to the intergenerational or personal traumas experienced in residential schools. Stakeholders working in the area of sexual exploitation in Canada in Sethi (2007) highlighted that past abuse and disconnection from family and community can make Indigenous women and girls more vulnerable to grooming strategies used by traffickers who offer them a sense of belonging and security.

Adverse experiences connected to involvement in child welfare were also noted as pathways into sex trafficking (Baird et al., 2019). An analysis of police files and child welfare data revealed that those who had experiences with this system were more likely to report childhood maltreatment, to abuse alcohol, cocaine, and crystal methamphetamine, and to have been recruited into sex trafficking younger and by a stranger, compared to those who were not involved with child welfare. Baird et al. (2019) also identified that recruitment and entrapment strategies used on their participants were most commonly “grooming strategies,” which included offerings of money, attention, and gifts, as opposed to “aversive strategies,” which included threats or violence; this was particularly the case for those who had been involved with child welfare.

#### Living and Working Conditions While Being Sex Trafficked

Four articles explored the living and working conditions of victim/survivors while they were being trafficked. Participants representing service agencies and advocacy groups supporting sex and/or labor trafficking victim/survivors in Hanley et al. (2006)’s study reported that the working conditions of sex trafficking victims frequently include low or no pay, long hours, and physical and sexual abuse. While many victim/survivors from Eastern Europe in McDonald and Timoshkina (2004, 2007) reported restrictions to their activities and movement, extreme financial exploitation, and threats or abuse from their managers, others had much more freedom, lived in comfortable apartments, and earned enough to send money back to their families. Morselli and Savoie-Gargiso (2014) used police electronic surveillance data to examine resource sharing and control within a “prostitution ring” in Montreal where the women’s experiences were consistent with sex trafficking according to “Canadian and American law” (p. 250). Threats, verbal abuse and emotional manipulation were observed in the network. However, some of the women, who had been recruited as a youth, had considerable control over resources within the operation and participated in the management of finances, information, and employment of other women in the network (Morselli & Savoie-Gargiso, 2014).

#### Needs of Victim/Survivors

Five of 14 studies reported findings on the needs of sex trafficking victim/survivors. Of these, three identified health needs, including mental health and addiction (Hanley et al., 2006; Nagy et al., 2020; Pashang, 2018), and four identified material needs connected to longer-term stability (Hanley et al., 2006; McDonald & Timoshkina, 2004, 2007; Nagy et al., 2020). Pashang (2018) identified the mental health challenges experienced by non-status/illegalized sex-trafficked female youth in the GTA; 90% of the youth were experiencing anxiety, 88% were experiencing insomnia, 88% were experiencing depression, and 77% had engaged in self-mutilation. Substance abuse, obsessive-compulsive behaviors, and psychosomatic illness were also noted (Pashang, 2018).

In addition to mental health care, victim/survivors and service providers in Nagy et al. (2020)’s study identified the need for emergency medical care and dental care for sex-trafficked women in Northern Ontario. While Nagy et al. (2020) and Pashang (2018) highlighted sexual health needs, with most illegalized trafficked female youth in Pashang’s study reporting having an STI, McDonald and Timoshkina's (2004) sample of sex-trafficked Eastern European women indicated very low rates and little concern about STIs. Overall, the women in McDonald and Timoshkina's (2004, 2007) studies did not express having unmet health needs despite rarely accessing health services.

Additional identified needs of victim/survivors were focused on longer-term stability and included: access to material goods such as financial support, housing, food, and hygiene (Hanley et al., 2006; Nagy et al., 2020); skills and professional development such as assistance in accessing education, language learning, and employment (McDonald & Timoshkina, 2004, 2007; Nagy et al., 2020); legal and immigration services (McDonald & Timoshkina, 2004, 2007); and security from traffickers (Nagy et al., 2020).

#### Gaps and Barriers in Anti-Trafficking Responses

Eleven studies identified gaps and barriers to addressing sex trafficking and the needs of trafficked persons (De Shalit et al., 2020; Hanley et al., 2006; Kaye et al., 2014; Matheson & Finkel, 2013; McDonald & Timoshkina, 2004, 2007; Millar & O’Doherty, 2020; Nagy et al., 2020; Olson-Pitawanakwat & Baskin, 2021; Pashang, 2018; Sethi, 2007). Some of the identified barriers were connected to issues around conceptualizing sex trafficking (De Shalit et al., 2020; Hanley et al., 2006; Kaye et al., 2014; Matheson & Finkel, 2013; Millar & O’Doherty, 2020). For example, there were disagreements around coercion and consent across stakeholders representing law enforcement, government offices, service agencies, and advocacy groups responding to sex trafficking in Vancouver; as one law enforcement representative pointed out, these conceptual disagreements likely contributed to the fragmented anti-trafficking response during the 2010 Vancouver Olympic Games (Matheson & Finkel, 2013). Ambiguous legal definitions, and sensationalized depictions of trafficking, were also identified as barriers to the identification of victim/survivors by law enforcement, and the subsequent prosecution of trafficking cases (Hanley et al., 2006; Kaye et al., 2014; Millar & O’Doherty, 2020). An overemphasis on criminal justice responses to sex trafficking, or the use of the criminal justice framing of sex trafficking in service provision, was also associated with creating barriers to service access for victim/survivors. The use of legal definitions in service organizations was described as excluding victim/survivors with more complex experiences of coercion (Hanley et al., 2006; Kaye et al., 2014). Key aspects of victim assistance are also mandated to government and law enforcement agencies, and services are often tied, either implicitly or explicitly, to trafficking investigations (De Shalit et al., 2020; Kaye et al., 2014). Stakeholders involved in an anti-trafficking response in Calgary, including government and law enforcement representatives, identified that framing the issue from a predominantly criminal justice perspective fails to adequately understand and address the needs of victim/survivors (Kaye et al., 2014).

The stigmatization and criminalization of sex work were identified as barriers to accessing help within five studies (De Shalit et al., 2020; McDonald & Timoshkina, 2004, 2007; Nagy et al., 2020; Pashang, 2018). De Shalit et al. (2020) describe the approach adopted by most representatives from agencies in Ontario providing health and social services to victim/survivors in their sample as “paternalistic,” with one representative reporting that their organization required women to withdraw from all sex work in order to receive support.

The criminalization of sex work posed particular challenges for immigrant/migrant women and girls. Sex trafficked women and female youth in McDonald and Timoshkina (2004, 2007) and Pashang (2018) who did not have legal immigration status in Canada were at risk of criminalization for both sex work and their illegalized status. Pashang's (2018) survey results revealed that many victim/survivors feared Canadian authorities more than their traffickers, and some preferred to remain underground rather than seek formal support and risk deportation. Language barriers and experiences of racism contributed to social isolation among trafficked immigrant/migrant women, and further limited their access to resources and knowledge of services (McDonald & Timoshkina, 2007).

The systemic oppression of Indigenous peoples was associated with other barriers to receiving help. Stakeholder participants working in the area of sexual exploitation in Canada commented that there is a lack of recognition of the issue of trafficking in Indigenous communities, and trafficking victim/survivors and their communities may not identify the situation as trafficking (Sethi, 2007). These stakeholders also noted that the police are reluctant to respond to sex trafficking that occurs within First Nations communities (Sethi, 2007). A lack of culturally appropriate and accessible services, especially in remote Indigenous communities, and a lack of collaboration between Indigenous communities and non-Indigenous policy makers and service agencies (Nagy et al., 2020; Olson-Pitawanakwat & Baskin, 2021; Sethi, 2007), were also identified as barriers to care. This was particularly true for sub-groups within Indigenous communities such as Two-Spirit people (Olson-Pitawanakwat & Baskin, 2021). Recommendations by service providers and participants with lived experience relating to sex trafficking included the use of holistic, non-stigmatizing, trauma and violence-informed approaches, and the use of Indigenous cultural teachings and healing ceremonies (Nagy et al., 2020; Olson-Pitawanakwat & Baskin, 2021)

#### Effectiveness of Interventions

Only one study in this review evaluated an intervention targeting victim/survivors of sex trafficking (Gow et al., 2015). This was a program evaluation of an outreach intervention using text messages to link potentially exploited sex workers advertising on Backpage.com to services in Edmonton, Alberta such as counseling, victim advocacy, and programs to receive education bursaries. The program staff received replies to 14% of their text messages, of which 77% were neutral in tone, 14% were negative, and 7% were positive. Although the impact of the program could not be rigorously evaluated, there were some instances of new service users at partnered agencies who reported having received the text messages. The authors conclude that two-way text messaging is a viable and promising means to provide social support to sexually exploited or trafficked women (Gow et al., 2015).

## Discussion

This review, is, to the best of our knowledge, the first to synthesize the existing empirical literature in Canada focused on the sex trafficking of adolescent girls and women. The results of this review reveal that empirical knowledge about sex trafficking of women and girls in Canada is sparse, thematically broad, and has many limitations. See Table 2 for a summary of the critical findings of this review.

**Table 2.** Critical Findings.

The literature is limited in scope and is divided on how to define and measure sex trafficking
Conceptualizing sex trafficking, particularly understanding the coercion-consent continuum and the difference between sex trafficking and sex work, is a focus of many studies
The pathways into trafficking and barriers to accessing help are often connected to systematic issues such as immigration policy, the colonization of Indigenous peoples, and factors associated with child welfare involvement
An overemphasis on criminal justice goals, as opposed to root causes and victim/survivors’ needs, is a common critique of existing anti-trafficking responses

Only 14 studies met the eligibility criteria for this review. These studies were largely set in Ontario and had exploratory aims. Interviews or focus groups were the most common data collection methods and those studies collecting primary data relied primarily on stakeholders as participants. This is consistent with Timoshkina and McDonald (2009)’s meta-synthesis which found exploratory stakeholder interviews to be the main data source of their included studies. The exploratory nature of many of the reviewed studies demonstrates that this body of research is still in its infancy, which poses a challenge for the development of evidence-based policy and responses. It is unsurprising that stakeholder participants are commonly sought for interviews, as recruitment of people with lived experience can be challenging and is associated with risks to them (Barrett, 2013). However, since the 2009 review, several published studies have also incorporated quantitative methods such as surveys and secondary analysis of police file data (Baird et al., 2019; Kaye et al., 2014; Millar & O’Doherty, 2020; Morselli & Savoie-Gargiso, 2014; Pashang, 2018), and there has been growth in the number of studies that include victim/survivor data (Baird et al., 2019; Gow et al., 2015; Millar & O’Doherty, 2020; Morselli & Savoie-Gargiso, 2014; Nagy et al., 2020; Olson-Pitawanakwat & Baskin, 2021; Pashang, 2018), indicating efforts to fill research gaps.

Challenges in conceptualizing sex trafficking were a prominent theme in this review as evident in the different definitions used by authors and tensions among perspectives provided by stakeholder participants in several of the studies (De Shalit et al., 2020; Hanley et al., 2006; Kaye et al., 2014; Matheson & Finkel, 2013; McDonald & Timoshkina, 2004). Lack of clarity and consensus in definitions may be associated with barriers to the development of data-driven anti-trafficking responses. Conceptual disagreements have led to inconsistent identification of victim/survivors and fragmented data collection and sharing across NGOs and government agencies in Canada and internationally (Barrett, 2013; Franchino-Olsen et al., 2022; Ibrahim, 2021). Moreover, as also noted at the 2018 National Summit and Regional Roundtables on Human Trafficking (Public Safety Canada [PSC], 2018), the narrowness of legal definitions specifically, frequently led to service access being contingent on sex trafficking investigations (De Shalit et al., 2020; Kaye et al., 2014).

Indeed, the prioritization of criminal justice goals is evident in current anti-trafficking strategies in Canada. Many anti-trafficking programs and policies are based on the assumption that increased criminal justice intervention in issues associated with sex traffickings, such as sex work or street gang activity, will prevent the harms associated with it (Durisin & van der Meulen, 2021; Goodale, 2019; Ontario, 2020). Funding is frequently directed toward anti-trafficking and organized crime task forces, training for law enforcement on recognizing and intervening in trafficking, and supporting trafficking prosecutions (Goodale, 2019; PSC, 2016). While legal protection from exploiters is a need identified by some trafficking victim/survivors, the focus on criminalization fails to meet other immediate and long-term needs identified through this review, such as housing, financial security, citizenship or residency status, and health needs.

Some individuals were identified in the findings of the included studies as being particularly at risk for experiencing certain types and pathways into sex trafficking, and for facing barriers to accessing help. Indigenous women and girls were highlighted as being particularly vulnerable to domestic sex trafficking; this was connected in several studies to intergenerational trauma from abuse experienced in residential schools and the economic and social impacts of colonization including disconnection and displacement of Indigenous communities and child welfare involvement (Nagy et al., 2020; Olson-Pitawanakwat & Baskin, 2021; Sethi, 2007). Child welfare-involved victim/survivors, some of whom were Indigenous, were also more likely to have experienced child maltreatment, to have their need for social connection and acceptance exploited during the recruitment process, and to have been trafficked domestically (Baird et al., 2019).

Immigrant/migrant women and girls were also reported to experience unique pathways into sex trafficking. Despite the popular images of cross-border sex trafficking, many immigrant/migrant victim/survivors in the reviewed studies shared that they had been trafficked only after arriving in Canada, largely due to vulnerabilities created by Canadian immigration policies. At the 2018 National Summit and Regional Roundtables on Human Trafficking, community agency representatives highlighted the need to address the link between policies contributing to the precarity of work for immigrant/migrant workers, and labor and sex trafficking risk; specifically, the single-employer visa rule for Temporary Foreign Workers was thought to enable exploitation and abuse by employers (PSC, 2018).

The findings of this review highlight the importance of addressing the upstream factors associated with sex trafficking, including social and economic displacement, racism and colonial violence, and immigration policy. Canada’s *National Strategy To Combat Human Trafficking 2019-2024* (Goodale, 2019) was developed with a focus on combating these upstream conditions and improving access to and quality of services for victim/survivors. However, given a lack of available evidence as determined in this review, it is unclear if and to what extent the *National Strategy*’s proposed actions can address the needs of victim/survivors and gaps in anti-trafficking responses. Although the *Strategy* commits to funding service interventions for victim/survivors, this review found only one published article evaluating the effectiveness of an intervention and, as it was an outreach intervention aimed at making contact with victim/survivors, it did not measure specific outcomes. The dearth of empirical research investigating the effectiveness of service interventions for victim/survivors of sex trafficking is an issue in the broader literature outside of Canada as well (Dell et al., 2019), posing challenges to policymakers in identifying where spending should be directed to improve services.

### Limitations of the Reviewed Studies

Past reports have identified that the body of literature on sex trafficking in Canada is limited by barriers to data collection associated with fragmented definitions and measures, the underground nature of trafficking, and the perceived risks of identifying oneself as a victim/survivor (Barrett, 2013). This review highlights that these issues are ongoing; several studies referenced limitations due to a lack of reliable prevalence data on sex trafficking (Baird et al., 2019; Kaye et al., 2014; Matheson & Finkel, 2013; Millar & O’Doherty, 2020). Issues in recruiting participants with lived experience of sex trafficking led several studies to instead collect qualitative data from stakeholders. Only four of the reviewed studies collected qualitative data from trafficked persons (McDonald & Timoshkina, 2004, 2007; Nagy et al., 2020; Olson-Pitawanakwat & Baskin, 2021; Pashang, 2018); these studies relied on convenience samples, and two had sample sizes of only 5 people (Nagy et al., 2020; Olson-Pitawanakwat & Baskin, 2021).

Only five studies provided quantitative demographic information about victim/survivor participants. While there was some diversity of participants reflected in the five studies, it is concerning that Indigenous victim/survivors were underrepresented (Baird et al., 2019; Millar & O’Doherty, 2020) considering the unmet needs that have been identified in this population (Barrett, 2013; NWAC, 2014). Additionally, only one study reported the inclusion of non-cisgender persons (Olson-Pitawanakwat & Baskin, 2021).

### Limitations of this Review

Non-English language articles were not included for review and a search of gray literature was not conducted. While the focus on scholarly research was fitting due to the paucity of existing reviews of this kind, including gray literature in this review could have resulted in additional observations about the breadth of knowledge available to policy makers and program developers. Additional limitations of this review pertain to the difficulty navigating the various conflicting definitions of sex trafficking. The use of a particular definition of sex trafficking was not a requirement for study inclusion in this review as we wanted to explore the use of the various existing definitions in the literature; however, this means that comparisons of findings across studies should be made with caution.

### Implications for Research

This review demonstrates that sex trafficking research in Canada is still limited in scope and is challenged by inconsistent definitions and a lack of reliable baseline data. This poses a barrier to understanding and measuring the problem and, additionally, to developing evidence-based responses. Collaboration across government agencies, NGOs, and impacted communities to develop standardized data collection measures that encompass experiences across the coercion-consent continuum could begin to address some of these gaps.

As Canada’s *National Strategy* recognizes the need to address the social and economic roots of sex trafficking, research funding and support should be directed to communities that are highly impacted. Additionally, while the *Strategy* outlines the government’s commitment to funding services and programs addressing the needs of victim/survivors, interventions for this population lack empirical support. Thus, agencies which provide these services and programs should be supported in conducting evaluations and rigorous research into the effectiveness of their interventions. See Table 3 for a summary of implications for research, policy, and practice.

**Table 3.** Implications for Research, Policy, and Practice.

Implications for research
• Address fragmentation of knowledge by improving collaboration among researchers, communities, and government agencies, and creating standardized data collection measures that encompass diversity in experiences of sex trafficking
• Conduct research on existing services and programs for victim/survivors to determine their effectiveness
• Direct funding to disproportionately impacted communities to conduct research and develop and evaluate programs
Implications for policy
• Focus policy and funding on initiatives that address root causes of sex trafficking and on provision of evidence-based services and programs for victim/survivors
• Address legislation that does not recognize the coercion/consent continuum in sex trafficking and that which makes sex workers and immigrants/migrants vulnerable to criminalization
Implications for practice
• Ensure services are available for those who do not meet the legal definition of sex trafficking
• Address stigmatization of sex work in programs supporting sex trafficked persons
• Improve collaboration across sectors and within relevant communities

## Conclusion

There is little scholarly empirical literature on the sex trafficking of women and girls in Canada; what does exist is largely exploratory qualitative research and is focused on conceptualizing the issue. The disagreements over conceptualizations and the use of narrow legal definitions were associated with barriers to addressing victim/survivor needs and some conceptions were associated with stigmatization and criminalization of sex work. The studies highlighted social and economic pathways into sex trafficking and identified criminalization of sex workers and immigrants/migrants, and the ongoing impacts of colonization as policy-level barriers to preventing sex trafficking and addressing the needs of victim/survivors. Future research should address the fragmentation of current data collection on sex trafficking and the lack of research on the effectiveness of existing programs supporting trafficked persons.

## Appendix A

Databases searched in ProQuest and Web of Science

### ProQuest

Canadian Business & Current Affairs Database, APA PsycInfo, Sociological Abstracts, International Bibliography of the Social Sciences (IBSS), Canadian Business & Current Affairs Database: Social Sciences, Literature Online, Worldwide Political Science Abstracts, Applied Social Sciences Index & Abstracts (ASSIA), Social Services Abstracts, Canadian Business & Current Affairs Database: Health & Medicine, Canadian Business & Current Affairs Database: Literature & Language, ABI/INFORM Collection, MEDLINE, APA PsycArticles, Coronavirus Research Database

### Web of Science

Web of Science Core Collection, BIOSIS Citation Index, BIOSIS Previews, Data Citation Index, KCI-Korean Journal Database, MEDLINE, Russian Science Citation Index, SciELO Citation Index, Zoological Record

## Appendix B

### Search strategy in Web of Science

(TS=(“sex* traffic*” OR “Forced prostitut*” OR “Juvenile prostitut*” OR “Domestic minor sex traffic*” OR “DMST” OR “CSEC” OR “sex* exploit*”)) OR (TS=(((“Human traffic*” OR (traffic* NEAR/4 (persons OR women OR girl* OR youth OR child* OR juvenile* OR minor*)))OR ((modern OR contemporary) AND slave*)) AND (sex* OR prostitut*))) OR (TS=((prostitut* OR (sex* NEAR/2 (work* OR indust* OR trade OR trading OR labor OR transaction* OR sell*))) NEAR/3 (coerc* OR exploit* OR manipulat* OR traffic*))) OR (TS=(Pimp* NEAR/5 (traffic* or coerc* or force* or exploit* or mantipulat* or child* or threat* or adolescen* or teen* or youth or minor))) AND (TS=(Canada OR Alberta OR “British Columbia” OR Manitoba OR “New Brunswick” OR Newfoundland OR Labrador OR “Nova Scotia” OR Ontario OR “Prince Edward Island” OR Quebec OR Saskatchewan OR “Northwest Territories” OR Nunavut OR Yukon OR Toronto OR Montreal OR Vancouver))
